# Bovine Colostrum: Its Constituents and Uses

**DOI:** 10.3390/nu13010265

**Published:** 2021-01-18

**Authors:** Raymond John Playford, Michael James Weiser

**Affiliations:** 1 Barts and the London School of Medicine and Dentistry, Queen Mary University of London, London E1 2AD, UK; 2 Department of R&D, PanTheryx Inc., Boulder, CO 80301, USA; mike.weiser@pantheryx.com

**Keywords:** nutraceuticals, gut repair, growth factors, injury

## Abstract

Colostrum is the milk produced during the first few days after birth and contains high levels of immunoglobulins, antimicrobial peptides, and growth factors. Colostrum is important for supporting the growth, development, and immunologic defence of neonates. Colostrum is naturally packaged in a combination that helps prevent its destruction and maintain bioactivity until it reaches more distal gut regions and enables synergistic responses between protective and reparative agents present within it. Bovine colostrum been used for hundreds of years as a traditional or complementary therapy for a wide variety of ailments and in veterinary practice. Partly due to concerns about the side effects of standard Western medicines, there is interest in the use of natural-based products of which colostrum is a prime example. Numerous preclinical and clinical studies have demonstrated therapeutic benefits of bovine colostrum for a wide range of indications, including maintenance of wellbeing, treatment of medical conditions and for animal husbandry. Articles within this Special Issue of *Nutrients* cover the effects and use bovine colostrum and in this introductory article, we describe the main constituents, quality control and an overview of the use of bovine colostrum in health and disease.

## 1. Introduction

Bovine colostrum (BC) is the first milk produced after birth and is a rich natural source of macro- and micro-nutrients, immunoglobulins, and peptides with anti-microbial activity and growth factors. There is strong evidence that BC is important for the nutritional and immunological support, growth, and development of the new-born calf. It is produced by the milk industry and commercially sold to promote general health and immune support. There is also increasing evidence that BC may be of value for the treatment of a variety of medical conditions in children and adults [1,2] and as a supplement for athletes to aid exercise performance and recovery [3,4]. Its use is not restricted to humans, with evidence supporting a role for its use in animal husbandry and the health and wellbeing of large animals and domestic pets [5,6,7,8,9]. This article provides an overview of the main constituents of BC, variation in BC constituents over time following birth, and an introduction to its use in maintaining health and treating disease. Readers interested in detailed aspects of individual applications of BC are referred to the companion articles on related topics that will be published early in 2021: “BC and Gastrointestinal Disease” (Chandwe K. and Kelly M.P.); “Effect of BC on Immune Function” (Ghosh et al); “Use of BC in Sports Medicine” (Davison G.); and “Pediatric Value of BC” (Caitlin V., Burrin D., Sangild P.T.).

## 2. Constituents of BC

BC contains similar nutrients to mature bovine milk, although the macronutrient profile and growth factor, immunoglobulins, and other immune factor content changes markedly from BC to mature milk.

### 2.1. Macronutrients and Micronutrients

#### 2.1.1. Proteins and Peptides

BC contains higher total protein content than mature milk, mainly due to higher levels of immunoglobulins and casein. Total protein concentration in BC constitutes about 15% of day one BC (weight/weight), falling to approximately 3% in mature milk. Protein constituents can be divided into two groups: whey proteins which are the soluble protein components, and caseins which are the insoluble proteins, with both components providing nutritional and bioactive properties. Casein is the predominant phosphoprotein that accounts for about 75% of proteins in cow milk and cheese, with αs1-casein being the predominant protein fraction of bovine milk [10]. Casein contains peptides with opioid-type activity that have been shown to decrease gastric emptying in rats [11]. Casein components have also been shown to affect immune activity when assessed using cell culture [12] and ex vivo methodologies [13]. Casein may also play a role in preserving the activity and aiding adsorption of other biologically active peptides by reducing their digestion by pancreatic enzymes, by means of functioning as a competitive substrate [14]. This action is similar to that reported for bovine trypsin inhibitor which protects IgGs, growth factors, and other biologically active proteins against proteolytic degradation within the gut. Bovine trypsin inhibitor is present in BC at about 100 times higher concentrations than mature milk [15]. Studies have demonstrated that the co-presence of casein partially protects epidermal growth factor (EGF) from digestion in humans [14], and that the stability and absorption of IGF-1 [16] is also improved. Casein also possesses other metabolic and protective effects including protective activity against experimental bacteraemia through increasing myelopoiesis [17]. Therefore, casein should not only be considered as an energy source but also as a factor that possesses immune-regulatory, antibacterial, and anti-inflammatory properties.

Whey proteins include immunoglobulins, lactoferrin, α-lactalbumin, β-lactoglobulin, lactoperoxidase, glyco-macropeptide and several growth factors, including the EGF-receptor ligand betacellulin [18]. α-lactalbumin is present in BC in high concentrations, comprising about one-quarter of the total protein content (40% of whey protein), with a high content of essential amino acids [19]. In addition to nutrient value, many whey proteins possess biological activity, some of which only become activated following exposure to acidification or partial digestion. Biological activities include influencing immune activity, reducing inflammation, and stimulating repair, e.g., α-lactalbumin has been shown to reduce gastric injury caused by ethanol in rats [20], and α-lactalbumin possesses anti-microbial and antitumor activity in addition to binding calcium and zinc ions [21]. In addition, hydrolysates of both casein and whey have been shown to interact with toll-like receptors involved in innate immune responses [22]. β-lactoglobulin comprises 162 amino acids (MW 18.4 kDa) and is a good source of essential amino acids but is also a major immunogen for subjects suffering from cow milk allergies. β-lactoglobulin is not present in human milk.

#### 2.1.2. Carbohydrates

Carbohydrates in BC include lactose, oligosaccharides, glycolipids, glycoproteins, and nucleotide sugars. Lactose forms the predominant saccharide in BC, comprising approx. 2.5% [23], which is lower than in mature bovine milk or in human milk [24]. Lactose can provide galactose and glucose to the liver in support of glycogen synthesis and storage [25]. Dietary oligosaccharides are present at approx. 1 g/L in BC, roughly double the levels in mature milk [26], and can act as prebiotics because many are not digested in the upper intestine, passing intact into the colon where they act as a metabolic substrate for colonic bacteria [27]. Oligosaccharides are divided into two broad classes, i.e., neutral, and acidic. Neutral oligosaccharides (also known as galacto-oligosaccharides) do not contain charged carbohydrate residues. In contrast, acidic oligosaccharides contain at least one negatively charged residue of N-acetylneuraminic acid (sialic acid) [28]. Sialylated oligosaccharides comprise the majority of the oligosaccharides in BC, and approximately seven-fold higher levels of sialyl-oligosaccharides are found in BC as compared to mature milk [26,29]. The predominant oligosaccharides in BC are 3′-sialyllactose (3′SL) and 6′-sialyllactose (6′SL), and in vitro work has demonstrated the ability of 3′SL to feed Bifidobacteria, which are important early colonisers of the human gut during infancy [30,31]. In addition to oligosaccharides, BC is rich in glycosylated proteins, which may have functional relevance by acting as prebiotics due to the saccharide component being cleaved by bacterial glycosidases, thereby influencing the gut microbiome. One of the predominant glycoproteins in BC is bovine glycomacropeptide (GMP) which has different glycosylated forms and is created via κ-casein proteolysis during digestion. Bovine GMP has bifidogenic abilities, as shown by its concentration-dependent growth promotion of the *Bifidobacterium longum* subspecies *infantis* [32].

#### 2.1.3. Fats and Lipids

BC contains approx. 7% fat, predominantly comprising milk fat globules. The lipid fraction contains multiple components of potential health relevance, including ω-3 and ω-6 polyunsaturated fatty acids, conjugated linoleic acid, short chain fatty acids, gangliosides, and phospholipids. Fatty acid constituents of BC are approximately 65–75% saturated, 24–28% monounsaturated and 4–5% polyunsaturated [33,34], and the predominant fatty acids are palmitic and oleic, accounting for 40% and 21% of total fatty acids, respectively [34]. The fat component of BC is often removed from commercial preparations to aid stability and processing; however, it is not biologically inert. For example, gangliosides and phospholipids are polar lipids found in the milk fat globule membrane and are involved in multiple functions such as neuronal development, binding of pathogens, and immune activation. For an excellent review on the biological effects of phospholipids in milk and BC, see Verardo et al. [35]. Additionally, evidence continues to build for the importance of palmitate in early life nutrition [36,37], and oleic acid has been linked to benefits ranging from immunomodulation to cardiovascular health [38].

#### 2.1.4. Vitamins and Minerals

BC contains fat-soluble (A, D and E) and water-soluble (B series) vitamins, which may be relevant for many metabolic processes including bone growth and antioxidant activity. Vitamin D has also been implicated in supporting immune system function and mental health [39]. Most vitamins are typically higher in concentration within BC as compared to mature milk, especially vitamins B_2_, B_12_, E, and D (Table 1). Compared to mature milk, BC is also rich in several essential minerals including calcium, copper, iron, zinc, magnesium, manganese, and phosphorus, (see [15,23,40]).

### 2.2. Bioactive Components

**Figure 1:**
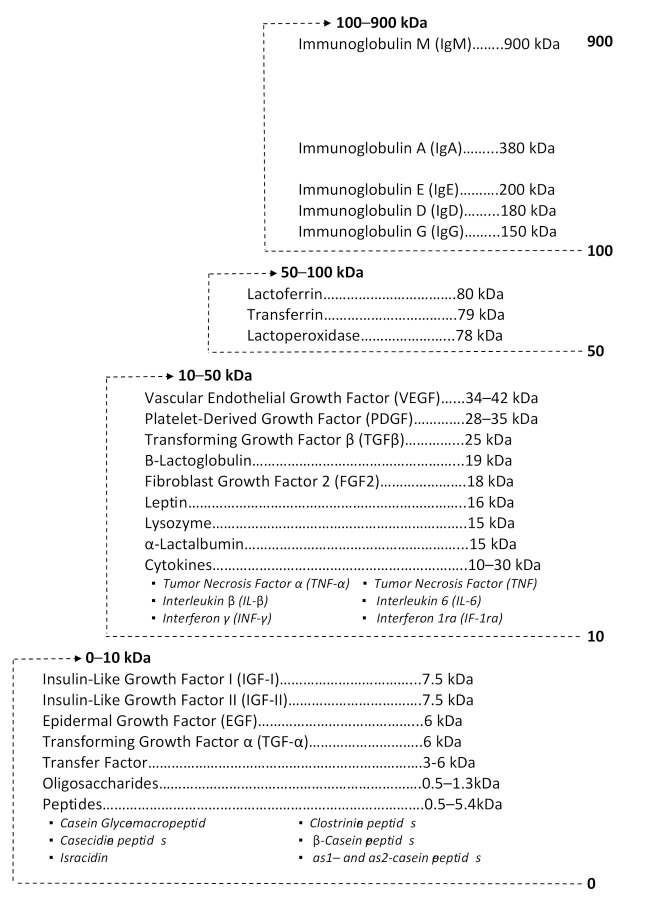
Molecular weight distribution for select components of Bovine colostrum (BC).

BC contains multiple components that influence the growth, development, and immune function of the suckling neonate, and cover a wide range of molecular weights (Figure 1). The major constituents of BC influencing these activities are discussed in the following sections.

#### 2.2.1. Antimicrobial Factors

##### Immunoglobulins

In mammals, immunoglobulins are important in passing passive immunity from mother to offspring. In BC, the major immunoglobulin is IgG, with a concentration of 30–87 g L^−1^, contributing approx. 80–90% of the total IgGs, with smaller amounts of IgA, IgD, IgE, and IgM being present (Table 1) [15]. In contrast to humans, where immunoglobulins can traverse the placental barrier, this does not occur in cows, and the calf’s sole natural source of immunoglobulins is from the consumption of BC. Immunoglobulins comprise a variable region that determine antigen binding specificity and a constant Fc region. Once an antigen is bound, immune complexes are formed, and the Fc region interacts with multiple immune effector cells, such as phagocytes, NK cells, dendritic cells and CD4+ T lymphocytes through binding to their Fc receptors. Bovine immunoglobulins can help prevent pathogen binding to host cells, present pathogens to macrophages for destruction, stimulate T cell and B cell immune activation, modify intestinal microflora, and induce local immunoglobulin A production [46]. IgG can, therefore, provide both passive immunity and modulate the adaptive and innate immune systems. Readers interested in the details of immune effects of BC and signalling pathways involved in microbe destruction and cytokine/antibody production, including IgFc interaction with effector cells, are referred to the upcoming article in this series on the “Immunological Effects of BC” by Ghosh S. et al. [46].

In addition to normal IgG constituents, specific vaccination of cows against human or bovine pathogens (hyper-immunisation) results in the production of neutralizing antibodies that show benefits for preventing and treating infections, resulting in increased weight gain in clinical and veterinary situations. Examples include preventing and treating enteropathic infections by *Escherichia coli* [47] or rotavirus [48]. Similarly, using a tooth surface model, a concentrate of hyper-immune milk was shown to prevent adherence of *Candida albicans* [49], and a hyperimmune BC preparation reduced dental plaque [50]. Although the use of purified specific antibodies from serum or milk appears to have value for infectious diseases [51], there may be additional benefits in using whole hyperimmune BC because it also enhances the repair process mediated through its growth factor constituents in addition to enhancing eradication of infections via its nonspecific antibacterial components.

##### Other Antimicrobials

**Figure 2:**
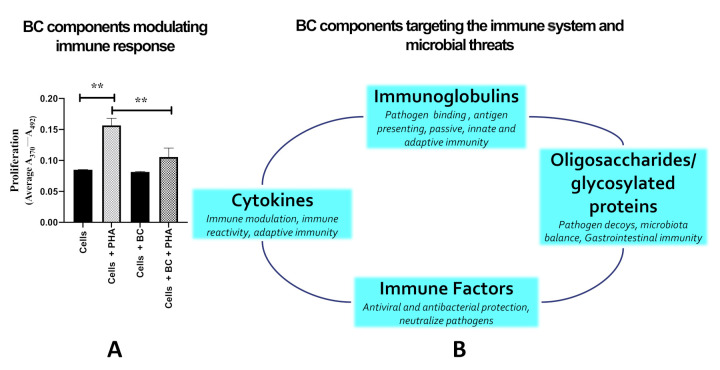
Influence of BC on immune function. (**A**) BC modulates immune response. BC reduced proliferation (BRDU incorporation) of human lymphocytes (*n* = 5 healthy subjects) stimulated by phytohemagglutination (PHA). PHA increased proliferation two-fold but was markedly truncated by BC (1 mg/mL). ** indicates *p* < 0.01 versus comparator. Data were kindly provided by Drs P Lalor and L Sheriff, University of Birmingham. (**B**) Multiple constituents of BC are involved in modulating the immune system and targeting microbes and other threats.

While immunoglobulins undoubtedly play an important role in preventing and resolving microbial infections, multiple other components of BC are also involved, as shown in Figure 2. Bioactive oligosaccharides present in BC may help protect against pathogens by acting as a prebiotic, i.e., stimulating growth of beneficial bacteria in the gut lumen. In addition, they may function as competitive inhibitors against toxigenic bacteria for binding sites on gut epithelial cells by mimicking epithelial cell surface carbohydrates [15,52]. For example, in vitro studies have shown that bovine milk oligosaccharides inhibit the adhesion of *E. coli*, *Salmonella*, *C. sakazakii*, and *H. pylori* [53].

Lactoperoxidase is an antimicrobial glycoprotein and member of the heme peroxidase family of enzymes that inhibits bacterial metabolism. Lactoperoxidase is an effective antimicrobial agent and is toxic to multiple Gram-positive and Gram-negative bacteria [54], through actions including the production of reactive oxygen species. These oxidative species can interact with certain amino acids in microbial proteins, leading to the inhibition of microbial metabolism and replication. Lactoperoxidase is added as a preservative in a variety of food products, cosmetics, and ophthalmic and oral hygiene products.

Lysozyme possesses antibacterial activity causing cell lysis of Gram-negative bacteria, as well as inhibiting the growth of Gram-positive bacteria [55,56]. Lactoferrin also enhances lysozyme antibacterial activity against *E. coli* [43]. Evidence in support of the value of lysozyme in BC also includes the finding that if lysozyme-deficit infant formulae is used, rather than one containing lysozyme, it results in a three-fold increase in diarrhoeal disease [57].

Lactoferrin is an iron binding glycoprotein (80 kDa) present in human colostrum and BC, although levels in BC are only about 10% of human values [58,59]. Lactoferrin induces multiple effects, including enhancing iron absorption as well as possessing antimicrobial activity [60,61], binding lipopolysaccharide, immune-modulating, and stimulating growth of intestinal epithelial cells and fibroblasts [62]. These findings suggest lactoferrin in BC may be relevant for the regulation of gut growth in neonates. Lactoferrin administration has been shown to reduce mortality and to increase growth in calves [55], reduce infections of the respiratory tract in bottle-fed infant humans [63], and reduce Giardia lamblia colonisation in children [64]. It also possesses immune modulatory activity, as demonstrated by reducing pollen antigen-induced allergic airway inflammation in a mouse model of asthma [65]. A Cochrane review also suggested that orally ingested lactoferrin may have value for reducing the risk of infections in addition to preventing the onset of necrotizing enterocolitis in preterm infants [66]. The concentrations of lactoperoxidase, lysozyme and lactoferrin in BC and mature milk are shown in Table 1.

#### 2.2.2. Cytokines and Immune Regulators

##### Cytokines

Immune defence is mediated through a combination of innate and adaptive processes. BC contains multiple constituents that may influence both of these pathways. The innate immune system is the first line of defence and protects against enteric infectious pathogens by detecting and eliminating them through cellular and molecular processes. These responses are subsequently progressed via the adaptive immune system, mediated through B and T cells. BC contains both specific immune factors (e.g., IgGs) and non-specific immune modulatory and antimicrobial factors (e.g., lactoferrin), with each component potentially having relevance for immune modulation. There is now extensive data showing that BC may have value for preventing and treating microbial infections, e.g., [67], working via the hosts immune function [68], in addition to attacking the microbe itself. As an example, volunteers given BC at the same time as receiving attenuated oral Salmonella typhi Ty21a vaccine produced increased levels of circulating specific IgA compared to controls who did not receive BC [69].

Cytokines are peptides/proteins involved in immune activation and recruitment, cellular signalling, and pathogen recognition. Cytokines are not usually involved in normal cellular homeostasis, but become relevant at times of stress, inflammation, or injury [70], stimulating actions such as differentiation, chemotaxis and protein synthesis. They regulate expression of a wide range of immune responses towards pathogens, influencing which types of immune cells are recruited for both adaptive and innate immune responses. BC and milk contain many cytokines, including TNFα, granulocyte, macrophage, and GMCSF and interleukin (IL) 1β, IL-6, IL-10 [55]. Cytokines likely play a role in modulating immunologic development in new-born and infants, working in combination with ingested maternal immunoglobulins and the nonspecific antibacterial factors in BC (Figure 2). Importantly, cytokines are not solely involved with stimulating inflammation, with some cytokines, such as IL-8, stimulating cell migration of colonic cell lines [71], and IL-10 being involved in preventing excess inflammatory response [72]. In support of the idea that BC dampens excess immune reactivity, a study using human colonic carcinoma cells (HT-29) showed that treatment with BC inhibited IL-1β-induced IL-8 expression, suppressed IL-1β-induced nuclear factor kappa beta (NFκβ) activation, and inhibited the degradation of inhibitor protein NFκβ [73], suggesting that BC may protect against intestinal inflammation by inhibiting the NFκβ pathway. Similarly, the presence of BC markedly reduces the phytohemagglutination (PHA)-induced proliferation of human peripheral lymphocytes (Figure 2A, previously unpublished data).

Although often considered as distinct, cytokines and growth factors have overlapping activities, e.g., the “cytokine” IL-8 stimulates the migration of human colonic epithelial cells [74], an action normally attributed to growth factors. Furthermore, there appears to be “cross-talk” between cytokines and growth factors in mediating their actions. For more detailed information on the effects of BC on immune function, readers are referred to the upcoming article in this Special Issue by Ghosh S. et al.

##### Other Immune Regulators

Fresh BC contains maternal leukocytes, such as B and T lymphocytes, macrophages, and neutrophils, in addition to epithelial cells [75]. These leukocytes protect the body against enteric pathogens by acting directly on the microbe, in addition to stimulating a local immune response, such as the production of cytokines, IgG, and antimicrobial factors [45]. The usual purification and preparation of BC for human use removes these cells from the final product.

MicroRNA elements with immune-regulating potential are also present in BC. These components are present in micro vesicles that are relatively stable during passage through the gastrointestinal tract and may influence lymphoid cell function within the intestine [76].

Colostrinin (also known as proline-rich polypeptide or PRP) is a naturally occurring mixture of proline-rich polypeptides derived from BC, which has been shown to help combat excessive inflammatory responses. The amino acid compositions of Colostrinin from ovine, bovine, and human colostrum are similar [77]. Colostrinin is not completely chemically defined, but comprises a mixture of at least 32 peptides with MWts 0.5 to 3 kDa [78], mainly derived from partial proteolysis of β-casein and aβ-casein. Colostrinin/PRPs help regulate the production of cytokines and may also inhibit the production of damaging reactive oxygen species [79]. Evidence in support of these actions include the findings that Colostrinin prevents allergic inflammation due to common and outdoor allergens in a murine allergic airway inflammation model [80], and immunocompromised rats infected with enterotoxigenic *E. coli* had reduced endotoxin levels and infected lymph nodes when treated with Colostrinin [81].

#### 2.2.3. Growth Factors

BC contains multiple components that stimulate growth, differentiation, and development. Although growth factors are normally considered to be peptides or small proteins, several other factors present in BC induce similar effects, although not usually acting via a classical receptor ligand interaction. These include glutamine, nucleotides, and polyamines and are sometimes termed preferred substrates, as opposed to growth factors. Nevertheless, these molecules play an important role in maintaining gut growth and immune activity, acting either directly or through altering the intestinal flora.

Over twenty different peptide growth factors have been described in BC, and the main ones are described in the following section. Although described individually, it is important to note that their functions are interrelated; cells are exposed to multiple factors at any one time and may result in additive or even synergistic responses. This was demonstrated by the finding that when bovine lactoferrin and EGF were added together to rat intestinal IEC-18 cells, it resulted in a synergistic growth response [82].

**Figure 3:**
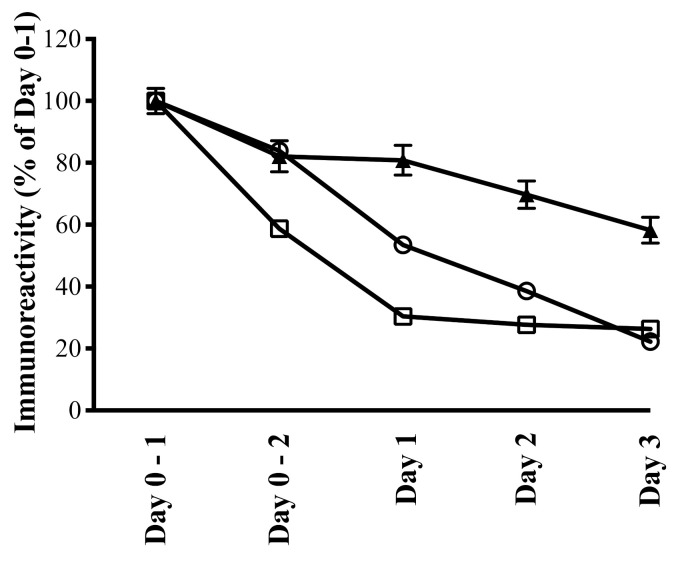
Change in IgG and some growth factor constituents within BC during the first few days post-calving. BC was collected at the first and second milking on day 0, and daily for the following 3 days from 6 cows post-calving, i.e., Day 0-1 and 0-2 samples were both collected on the first day after calving. Samples were analysed for IgG and growth factor concentrations using commercial ELISA kits. Immunoreactivity is expressed as % of Day 0-1 sample. EGF (▲), IGF1 (□) and IgG (○). Results are expressed as mean ± SEM of 6 animals per time point, with each sample measured in triplicate. The concentrations of all constituents shown were significantly reduced in each subsequent collection, *p* < 0.01 vs. their Day 0-1 value. Adapted from [83].

Growth factor constituents of colostrum vary markedly across species, for example, EGF content of human colostrum is much higher than BC. Even within species, major changes occur during the first few days post-birthing—some studies have shown marked reductions in multiple growth factor constituents of BC during the first 48–72 h post-calving (Figure 3 [83]).

##### Insulin-Like Growth Factors (Somatomedins) and Their Binding Process

IGF-I and IGF-II promote cell proliferation and differentiation [84], and there is 100% sequence homology between the bovine and human IGFs. They have structural similarity to pro-insulin and, when administered at high concentrations, IGFs can exert insulin-like effects. Endogenous IGF is mainly produced by the liver [85]. The concentration of IGF-I within BC is much higher than that found in human colostrum (500 mg L^−1^ versus 18 mg L^−1^) [86,87], with IGF-I levels falling to approx. 10 mg L^−1^ in mature bovine milk [88]. IGF-I and-II have been shown to survive exposure to both acid and heat, and it is therefore likely that they remain biologically intact during commercial milk processing as well as during passage through the stomach [89]. IGF-I is an anabolic factor promoting protein accumulation [90], and is probably involved in mediating the growth-promoting actions of growth hormone.

Within BC and human colostrum, IGFs exist in both bound and free forms. Free IGF levels vary during the perinatal period, with the majority of IGF-I in BC being present in the free form, whereas the reverse is true in the antepartum period and in mature milk [91]. Six IGF binding proteins (IGFBPs) have been identified, and one of their functions is to act as carrier proteins, reducing the proteolysis of IGF during its transit through the intestine. Importantly, modulation of IGF-I activity can be mediated by changes in the relative percentage of IGFs present in the bound state; proliferative activity is only seen in the unbound IGFs. In addition, some studies suggest that there is cross-talk between the gut microbiome and circulating IGF levels [92].

The high concentrations of IGFs in BC suggest that they may be important in mediating local gut anabolic-reparative effects. There has been debate however, regarding whether IGFs within BC are absorbed intact, raising the theoretical concern that this might increase the risk of prostate cancer, because prostate cancer cells may express IGF-I receptors. However, the pathophysiological situation is more complicated, because administration of IGF-I to noncancerous prostate cells increases differentiation [93]. It is therefore reassuring that one study measuring IGF levels in subjects taking high doses of BC (40 g/day for several months) showed no significant increase in plasma IGF levels [94].

Epidermal Growth Factor Receptor Ligand Family

This family of peptides all bind the EGF receptor (also known as the c-erb1 receptor), and include EGF, TGF-α, betacellulin, mammary-derived growth factor II, and human milk growth factor III. Other related members of this EGF-receptor binding family that are not present in significant concentrations in BC are heparin binding EGF and amphiregulin (for a detailed review of this family of peptides see [95]).

##### Epidermal Growth Factor

EGF contains 53 amino acids and is produced by the salivary glands and Brunner’s glands within the duodenum. EGF is present in human colostrum (200 mg L^−1^) and milk (30–50 mg L^−1^), as well as in many other species. Studies examining the stability of milk-borne EGF by incubating with gastric juice from preterm infants suggests that it remains intact [96], whereas adult gastric juice causes a major reduction in the bioactivity of EGF due to it being cleaved to an EGF1-49 form [97]. The relative stability of EGF within the small intestine is dependent on whether additional food proteins are present. In fasting conditions, it is rapidly destroyed by pancreatic proteolytic enzymes, whereas if ingested food proteins such as casein or trypsin inhibitors are also present, the EGF is preserved [14]. The EGF receptor is restricted to basolateral membranes and not present on apical (luminal) surfaces of the gut [98]. This suggests that orally ingested EGF-receptor ligands would not affect the normal adult intact gastrointestinal tract but would be immediately available to stimulate repair at sites of injury where the basolateral receptor becomes available, acting as a “luminal surveillance peptide”, immediately available to stimulate the repair process at sites of injury [99].

It is important that distinction between the physiological effects of oral EGF-R ligands in adults and neonates is made. The presence of EGF as a constituent of human colostrum or in BC within the gut lumen may be able to access basolateral receptors within the immature neonatal gut [100] due to its increased permeability, and could, therefore, help prevent bacterial translocation [101] and stimulate gut growth in suckling neonates. Interestingly, an alternative approach to locally deliver EGF and reduce its proteolytic digestion involved administering *E. coli* bacteria that had been genetically modified to produce human EGF. This was seen to be beneficial in reducing dextran sodium sulphate (DSS)-induced colitis in mice [102].

##### Transforming Growth Factor α

TGF-α concentrations in human colostrum and milk are much lower concentrations (2.2–7.2 mg L^−1^) than EGF [103]. TGF-α contains 50 amino acid residues and is produced by mucosa of the entire length of the gut [104]. Systemic administration of TGF-α has multiple effects including reducing gastric acid production, increasing gastric mucus production, and stimulating gut growth and repair [105]. However, some of these effects are probably pharmacological, rather than physiological, because TGF-α expression in the small intestine is restricted to the superficial (non-proliferating) areas, suggesting that its physiological function could be related to cellular migration and differentiation as opposed to cell growth.

##### Transforming Growth Factor β Family

There are 35 isoforms of TGF-β, and this group of peptides have a distinct structure from that of TGF-α. In most proliferation assays, the TGF-β family inhibits proliferation, rather than stimulating it. They are mainly expressed in the superficial zones of the normal gastrointestinal tract. TGF-β has several functions, such as acting as a chemoattractant for neutrophils and increasing epithelial cell migration at the edges of wounds [106]. TGF-β is therefore probably important in stimulating the initial stages of repair, where surviving cells at the wound edge migrate over the damaged region to re-establish a continuous epithelial layer. Concentrations of TGF-β within BC are high (20–40 mg L^−1^), and although they are lower in mature milk, they still are at relatively high levels (1–2 mg L^−1^). These levels are sufficient to protect rat stomachs against indomethacin-induced injury [107], suggesting that TGF-β in BC (and human colostrum) may be important in maintaining gastrointestinal integrity in suckling neonates [108]. For an excellent review on the roles of TGF-β on gut, skin and lung integrity see [109].

##### Platelet-Derived Growth Factor

As the name suggests, platelet-derived growth factor (PDGF) was initially identified in platelets, however it is also produced and secreted by macrophages. PDGF is acid-stable, consisting of two disulphide-linked polypeptides: chain A (14 kDa) and chain B (17 kDa). The dimer exists in three isoforms (AA, AB, and BB) that bind tyrosine kinase-type receptors. PDGF stimulates proliferation of a variety of cells including fibroblasts and arterial smooth muscle cells, and oral administration of PDGF enhances ulcer healing in animal models. Although human colostrum and BC contain PDGF, the majority of PDGF-like proliferative activity in BC is actually due to bovine colostral growth factor, which shares sequence homology with PDGF [110,111,112].

##### Vascular Endothelial Growth Factor

Vascular endothelial growth factor (VEGF) is a heparin-binding glycoprotein and is present as a homodimer (Mwt 34–42 kDa). VEGF exhibits many actions of potential pathophysiological significance, including stimulating proliferation and new vessel formation and vascular permeability-enhancing activity [113]. VEGF is present in human breast milk at about 75 mg L^−1^ during the initial seven days of lactation, falling to about 25 mg L^−1^ in the following week [114]. Due to its angiogenic activity, the VEGF content of BC may have value in enhancing local vascular supply in conditions such as peptic ulceration.

##### Milk Fat Globule-Epidermal Growth Factor 8 (MFG-E8)

Milk fat globule-epidermal growth factor 8 (MFG-E8) is a secreted protein initially identified as a critical component of the milk fat globule. It is present in BC in high concentrations and may influence the immune and repair response of the suckling neonate [115]. MFG-E8 enhances the removal of damaged and apoptotic cells by phagocytosis, induction of VEGF-mediated new vessel formation, and enhancement of mucosal healing [116].

#### 2.2.4. Hormones

BC contains multiple hormones, including prolactin, somatostatin, oxytocin, luteinizing hormone-releasing hormone, thyroid-stimulating hormone, thyroxine, calcitonin, oestrogen, and progesterone. It is probable that at least some of these factors influence development of the suckling neonates [117], due to passage of the hormones from the gut into the circulation. This is less likely to be relevant in adults, because the reduced permeability of the adult gut restricts the passage of most of these factors, as demonstrated by the finding that ingestion of large amounts of BC failed to increase plasma IGF-1 levels in normal adults [94]. However, it remains possible that systemic absorption could have relevance in disease states where gut permeability may be increased.

##### Growth Hormone and Its Releasing Factor

Growth hormone (GH), its releasing factor (GHRF), and binding protein are all present in human colostrum, BC, and milk [118]. Suckling neonates have high circulating levels of GH, probably due to the consumption and absorption of GH as well as GHRF, which causes the neonate to produce endogenous GH from the pituitary gland [119]. The proliferative effects of GH are mediated partly by GH itself and partly due to the stimulation of IGF-1 production and secretion [120]. Systemic GH probably plays a role in gut growth and function, with GH receptors present throughout the human gastrointestinal tract [121]. In addition, studies have suggested that there is cross-talk between GH, IGF-1, and the gut microbiome [122]. However, the effect of GH within the gut lumen (as occurs when ingesting BC) is uncertain, because stimulation would probably only occur if the GH receptors were present on the apical surfaces of the enterocytes, which is currently unknown.

##### Leptin

Leptin contains 167 amino acid residues and has an MWt of 16-kDa. This hormone is important in controlling energy intake and expenditure, through actions on appetite and metabolism. Leptin acts on hypothalamic receptors in the brain, inhibiting appetite by counteracting actions of the feeding stimulators neuropeptide Y and anandamide and by stimulating synthesis of the appetite suppressant α-melanocyte-stimulating hormones. Although leptin is only present in BC at low concentrations (13.9 mg L^−1^ [123]), a study in a diabetic mouse (ob/ob) strain showed that only very low concentrations of leptin are required to exert effects on glucose metabolism [124].

## 3. Assessment of BC for Human Use

BC is widely available for human and animal consumption from health food stores and via the internet. BC is normally collected and frozen on the individual farms and shipped frozen to central processing facilities, where it undergoes pasteurisation, optional defatting and the removal of lactose, then spray- or freeze-drying to a powder. This powder product may then be sold in powder form or subsequently incorporated into dietary supplement formats such as sachets, capsules, chewable tablets, and soft chews. There are some food categories where BC has been, or could be, utilised as a functional ingredient, such as cheeses, jellies, yoghurts, ice cream, nutritional bars, milk powdered beverages, and ready-to-drink beverages.

BC routinely undergoes high temperature short duration pasteurisation (usually about 15 seconds at 72 °C) or alternatively batch pasteurisation, where the BC is heated to between 60 and 63 °C for anywhere between 30 min and 60 min [125]. Once dried, BC powder is normally recommended to be stored at room temperature, and most commercially available powders have at least one-year shelf life, in some cases longer.

The majority of BC for human use and for clinical trials is either complete BC (containing the fat component) powder or the defatted (skimmed) powder. Other preparations include the sterile filtering of raw BC, BC which has had subfractions removed (such as casein), or a combination of selective removal of constituents such as casein and lactalbumin, combined with the enrichment of factors such as immunoglobulins and growth factors, e.g., as used in [2]. It is, therefore, important that the form of BC is noted, in addition to the dosage being used.

IgG content of BC is considered a surrogate marker of quality, with concentration > 50 g/L in fresh BC generally being considered acceptable [126,127]. IgG content of BC will vary according to the breed of cattle, age of the animals, and other factors including the feeding regimen, but one of the most important factors is the timing of the BC collection post-calving, with IgG levels (along with many of the growth factor constituents) falling rapidly on day 2–3 after calving (Figure 3) [83]. Although there is no formal definition regarding how many days following calving the product can be classified as colostrum rather than milk, it is generally accepted BC should only be considered as such up to day 3 post-calving [128]. However, some producers do market “late colostrum” which is collected between days 5–7 post-calving.

In parallel with the fall off in IgG and growth factor content, studies examining the biological activity of BC collected on day 1 post-calving versus day 3 have demonstrated major differences in their ability to stimulate the growth and repair of human gut cells in in vitro models of gastric damage [83] and in reducing increased gut permeability in humans [129]. However, differences in the BC collection period are not the only cause of bioactive variability; variation in storage conditions, processing, and pasteurisation may also contribute to the six-fold difference in bioactivity seen when commercial products were compared [83]. These wide variations in bioactivity are of particular concern if BC is being used as a therapeutic agent for a medical condition, where consistency of the product is vital.

### Use of BC in Combination with Other Nutraceuticals

Although the focus of this article relates to use of BC as a single immunomodulatory/protective agent, several products are commercially available that comprise BC in combination with one or more additional components and are sold as supplements or foodstuffs. These additional components include proteins, egg, carbohydrates, vitamins, probiotics, and plant polyphenols. Some combinations have been examined using crossover randomised trials to establish additive or synergistic effects. Examples include reports showing synergistic effects, when BC and zinc carnosine were combined for reducing exercise induced raised gut permeability [130], and when BC was combined with chicken eggs in promoting the growth and repair of human gut cell lines and in reducing DSS-induced colitis in mice [131]. However, most combination products have not undergone vigorous testing to show the benefit of using more than one nutraceutical. Readers interested in the use of combination products for sports nutrition are referred to [132] and the upcoming article in this Special Issue on the “Use of BC in Sports Medicine” by Davison G.

## 4. BC Use in Human and Veterinary Health

Separate articles to be published within this Special Issue will cover the relevance of BC for use in gastrointestinal damage (see Chandwe K. and Kelly M.P.), immunology (see Ghosh S. et al.), paediatrics (see Caitlin V., Burrin D. and Sangild P.) and sports medicine (see Davidson G. et al.). These will, therefore, only be covered briefly in this article.

### 4.1. BC Therapy for Human Gastrointestinal (GI) Health and Disease

**Figure 4:**
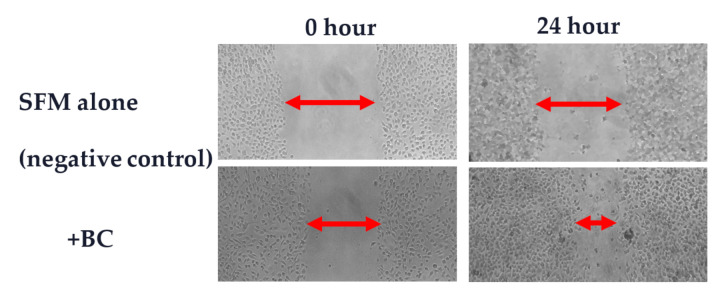
Effect of BC on cell migration. Human colonic Caco-2 cells were grown as a continuous monolayer and a standardised wound produced at time 0. The amount of closure of wound was assessed 24 h later. The presence of BC significantly increased the rate of closure compared to cells grown in serum free medium (SFM, negative control) alone.

Whatever the initiating injury, denuded areas of the gut undergo repair via standard processes. Rapidly after injury, surviving cells at the wound margin migrate across the denuded region to re-form a continuous epithelial layer, a process termed restitution. Twenty-four to forty-eight hours later, there is increased proliferation to replenish lost cells. BC stimulated both cell migration and proliferation when tested against multiple human and rodent intestinal cell lines. A typical pro-migratory response of a gastrointestinal cell line to the presence of BC is shown in Figure 4. In addition, the immune modulatory components within BC are probably also relevant in limiting excess inflammatory responses (as shown in Figure 2).

BC shows therapeutic potential for a variety of GI conditions, including nonsteroidal anti-inflammatory drug (NSAID) gut injury [2], short bowel syndrome, chemotherapy induced mucositis (particularly oral mucositis) [133], and inflammatory bowel disease [134,135]. In addition, preclinical studies using BC or its hyperimmune variants show promise for several gastrointestinal infectious diseases, such as Clostridium difficile [136]. The mechanisms of action of BC in these conditions will be discussed in detail in the accompanying review articles on the effect of BC on gastrointestinal injury, paediatrics, and immune function, but one potential mechanism of action, relevant for all these situations, may be through modulating the gut microbiome. It is well established that breast feeding of human infants results in a gut microbiota dominated by species of Bifidobacterium and with decreased Enterobacteria, compared to formulae-fed infants. These differences are likely to be due, at least in part, to differences in oligosaccharides and other prebiotic factors present in human and formula milk [137]. Many of the GI conditions mentioned above, such as NSAID-gut injury, necrotising enterocolitis, and inflammatory bowel disease, have been shown to have associated dysbiosis, and several animal models of GI disease have demonstrated that BC favourably alters gut microbiome as well as improving healing, suggesting a causal link [138]. However, the evidence from human clinical trials that the gut microbiome is significantly altered by BC administration, or is an important mechanism in mediating repair, is less clear, and further studies are required. For example, one study examining the effect of egg and BC on children with growth stunting did not find any substantial difference in the 16S configuration of the faecal microbiota between children receiving BC/egg and the control group, although there was an increase in *Streptococcus thermophilus*, which may function as a probiotic [139]. BC is also likely to directly affect the gut mucosa, and studies on animal models and human subjects suggest the BC can reduce apoptosis in damaged areas through actions on Caspases, bcl-2 and HSP70, in addition to strengthening tight junctions through actions on occludin, claudin and zonulin [130,140].

### 4.2. BC and Immune Function in Health and Disease

The immune components of BC offer potential for a range of conditions. It is unlikely that the bovine IgGs are absorbed into the human systemic circulation intact, but there is mounting evidence that ingestion of BC can influence immune function outside of the GI tract. Examples include findings that BC supplementation reduced the number of flu-like episodes [141]. BC supplementation has also been reported to be beneficial in reducing the number of upper respiratory tract infections (URTIs) and diarrhoeal episodes in children [142,143], and BC IgG has been shown to bind and neutralise human respiratory syncytial virus [144]. Similarly, athletes undergoing training are known to have increased risk of URTI symptoms, and metanalyses of published trials reported a significant positive effect of BC supplementation [145]. Additional studies examining the efficacy of BC to enhance immune function in different age groups and influence sensitivity to URTI and other infections would be of value. Interested readers are referred to the companion articles currently being published on the “Effect of BC on Immune Function” (Ghosh et al) and the “Use of BC in Sports Medicine” (Davison G.).

### 4.3. BC and Skin

Although there are only limited data, in vitro studies have shown that BC induces proliferation and differentiation of skin [146] and stimulates repair and reduces artificially induced inflammation in animal models [147,148]. Subcomponents of BC have also shown beneficial effects; for example, topical administration of lactoferrin reduced inflammation in human volunteers exposed to local skin allergens [149].

### 4.4. BC and Bone Density

Animal models suggest that BC supplementation may have value in increasing bone density in juvenile rats [150]. There are limited randomised human clinical trials examining the effect of BC on bone density, although one study reported BC supplementation during resistance training increased both leg press strength and truncated bone resorption in older adults [151].

### 4.5. BC, Diabetes, Hypercholesterolemia, and Non-Alcoholic Fatty Liver Disease (NAFLD)

Several studies in animals and humans suggest that BC may have value in reducing raised glucose and lipids in Type-2 diabetic subjects [152,153], patients with hypercholesterolemia, and/or suffering from NAFLD [154,155]. All these conditions may occur in the same patient and have similar pathophysiological processes. These include relative insulin resistance, low grade chronic inflammation in multiple tissues including fat and increased gut permeability, possibly due to the disruption of intestinal intercellular tight junctions contributed to by the presence of gut luminal bacterial lipopolysaccharides. The beneficial effects of BC for these conditions are likely to be due to multiple constituents that affect interaction with luminal bacteria and lipopolysaccharide (LPS), mucosal integrity, and innate and adaptive immune responses. Some of these mechanisms are shown in Figure 2B. Evidence in support of BC influencing the patients’ immune responses in these conditions include findings that BC administration decreases serum tumour necrosis factor (TNF)-α levels, in addition to increasing the number of splenic NKT cells [156] and circulating CD4+ CD25+ HLA-DR T_reg_ cells [155]. Readers interested in a detailed review of the immune effects of BC are referred to the upcoming article by Ghosh S et al.

### 4.6. BC in Veterinary Practice

Calf survival and postweaning morbidity and mortality are significantly reduced if the new-born calf receives BC within the first few hours after birth [126]. It is therefore important that new-born calves receive sufficient BC during this period. Large amounts of BC remain available that are surplus to the calf’s requirements, and this is collected and processed as described earlier. The use of BC in veterinary practice falls outside the usual scope of *Nutrients*, and this section, therefore, only briefly reviews some of the evidence for benefit of BC use in both production and companion animals. Interested readers are referred to the specialist articles cited.

#### 4.6.1. Use of BC in Production Animals

For calves, early feeding of BC within the first hours of life is critical for their health and survival. General guidelines are a first feeding of approximately 10% of the calf’s weight in BC. This early feeding provides the immunoglobulins the calf requires to enable passive immunity during the first 24 h prior to gut closure, during which time the immunoglobulins can reach the calf’s circulatory system [15]. Early BC feeding increases daily weight gain during the calf’s growth period and beneficial effects are maintained long-term, with the adult dams who had been fed BC as a calf producing increased milk yields. [157]. Furthermore, calves who were not given BC had a 74-fold higher likelihood of mortality by 21 days of life than those who received more than two quarts of BC within the first 6 h of postnatal life [158]. The beneficial effects of BC ingestion in cows are likely to be due, at least in part, to raising serum (and gut luminal) IgG, enhancing the calf’s ability to fight infection. Other factors that may also be involved include altering immune responses via cytokine constituents, altering the faecal microbiome, and anabolic effects of hormones and growth factors within BC on the gut and in other tissues.

For piglets, the postweaning period is a critical time, where susceptibility to environmental pathogens and gut-associated pathologies can impact the production pig’s size and survival. For example, the piglet small intestine loses 20–30% of its weight in the first two days post-weaning [159]. In this same study, BC administration was shown to enhance weight gain and systemic IgA response [159]. Similarly, in piglets weaned at 21 days who received BC for two weeks following weaning, there were beneficial effects on intestinal morphology, including increased villi height, decreased crypt depth, and increased epithelial cell height in the small intestine [160]. This beneficial effect of BC in piglets is further supported by studies showing a positive effect of BC on average daily food intake and weight gain during the first week of the postweaning period [161]. Mechanisms of action of BC in pigs are likely to be similar to those for use in cattle.

For poultry, feed conversion efficiency is important for broiler farms, because the reduction in feed needed during the growth and finishing periods greatly impacts costs. A trial of BC supplementation (5% by weight) into the feed of broiler chicks for the first two weeks after hatching showed greater feed conversion efficiency in the BC group [162]. Similarly, BC supplementation to young broilers (1–10 days old) under heat stress showed benefits on thigh and breast size compared to the control group [163].

#### 4.6.2. Use of BC in Canine and Equine Animals

In canines, studies have suggested benefits for the use of BC for gastrointestinal health, digestion, and immune support. In puppies, a study on recently weaned toy breed puppies found that 0.5 g of BC per day for ten days improved faecal quality, as measured by the WALTHAM faecal scoring system, compared to puppies receiving milk powder as a control [164]. Additionally, in published abstract conference proceedings [165], a pilot crossover trial supplemented a group of neutered male beagles with 1 g of BC per day for three weeks, in addition to a set of four probiotic strains at 2.9 × 10^9^ colony forming units. They did not observe changes in faecal microbiota, but did see improvements in protein digestibility in the BC-supplemented group. In another study, supplementation of 2–7-year-old huskies with BC for 40 weeks increased faecal IgA levels compared to controls, suggesting enhanced gut-associated lymph tissue function. In addition, they responded to canine distemper virus (CDV) vaccination with higher plasma levels of anti-CDV IgG, and had increased faecal microbiota diversity, suggesting positive effects on immune function and gut microbiome [7].

For horses, it has been shown that immunoglobulins from BC can be absorbed by new-born foals, with the bovine IgG remaining in the circulation with a half-life of seven days [166]. The beneficial effect of BC ingestion by horses is also supported by findings that thoroughbred yearlings who received BC had two-week shortening of illness due to respiratory disease when compared to control yearlings during the 22-week supplementation period [6]. Furthermore, in a randomised cross-over trial, racing thoroughbreds earned more race purse money, ran faster races, and were able to return to racing a week faster during the period they were supplemented with BC compared to the period when they were not [5]. To assuage concerns about potential increases in serum growth factor levels, the same group completed a trial confirming that 200 grams per day of BC powder did not increase serum IGF-1 concentrations after two and four weeks of supplementation in thoroughbred horses [167], which is consistent with human studies discussed earlier.

## 5. Conclusions

BC is a rich source of macro- and micro-nutrients, immune modulators (including IgGs), growth factors, and other bioactive molecules. Current farming methods enable the production of large volumes of BC for clinical and veterinary use. Batch variation during production must be kept to a minimum to ensure reproducible content of constituents, and bioactivity and quality control procedures need to ensure that the processing and storage methods used are optimised to maintain stability. In the general health support market, BC has several advantages over single ingredient supplements; it is perceived as being a comprehensive “superfood”, with customer appreciation of its links to natures first food from breastfeeding. BC has a strong safety profile, relevance across all age groups, and is delivered in a natural formulation that limits its own inactivation when taken orally. These aspects should facilitate greater consumer and patient acceptance and compliance to achieve optimal immune and digestive health. In addition to administering BC on its own, additional therapeutic value may be gained if the BC is specifically tailored for individual conditions, e.g., administering hyperimmune milk or BC to immunocompromised patients who have gut disease, thereby addressing the problem of the gut infection while also enhancing gut repair. BC efficacy may also be increased if administered in combination with other factors that act synergistically with BC.

## Figures and Tables

**Table 1:** Concentrations of select macronutrients, micronutrients, immunoglobulins, and general antimicrobial peptides present in bovine colostrum (BC) and mature milk.

Component	BC	Mature Milk
Total solids (%)	24–28	12.9
Fat (%)	6–7	3.6–4.0
Protein (%)	14–16	3.1–3.2
Casein (%)	4.8	2.5–2.6
Albumin (%)	6.0	0.4–0.5
Total immunoglobulin (mg/mL)	42–90	0.4–0.9
Lactose (%)	2–3	4.7–5.0
**Minerals**		
Calcium (g/kg)	2.6–4.7	1.2–1.3
Phosphorus (g/kg)	4.5	0.9–1.2
Potassium (g/kg)	1.4–2.8	1.5–1.7
Sodium (g/kg)	0.7–1.1	0.4
Magnesium (g/kg)	0.4–0.7	0.1
Zinc (mg/kg)	11.6–38.1	3.0–6.0
**Vitamins**		
Thiamin (B1) (µg/mL)	0.58–0.90	0.4–0.5
Riboflavin (B2) (µg/mL)	4.55–4.83	1.5–1.7
Niacin (B3) (µg/mL)	0.34–0.96	0.8–0.9
Cobalamin (B12) (µg/mL)	0.05–0.60	0.004–0.006
Vitamin A (µg/100 mL)	25	34
Vitamin D (IU/g fat)	0.89–1.81	0.41
Tocopherol (E) (µg/g)	2.92–5.63	0.06
**Immunoglobulins**		
IgG1 (g/L)	34.0–87.0	0.31–0.40
IgG2 (g/L)	1.6–6.0	0.03–0.08
IgA (g/L)	3.2–6.2	0.04–0.06
IgM (g/L)	3.7–6.1	0.03–0.06
**Antimicrobials**		
Lactoferrin (g/L)	1.5–5	0.02–0.75
Lactoperoxidase (mg/L)	11–45	13–30
Lysozyme (mg/L)	0.14–0.7	0.07–0.6

Ranges are shown where available. Values were obtained from refs [15,23,40,41,42,43,44,45].

## Data Availability

Articles are licensed under an open access Creative Commons CC BY 4.0 license, meaning that anyone may download and read the paper for free.

## Abbreviations

DSS, dextran sodium sulphate; EGF; epidermal growth factor; EGFR, epidermal growth factor receptor; Ig, immunoglobulin; IGF, insulin-like growth factor; MDGF, milk-derived growth factor; MFG-E8, Milk fat globule epidermal growth factor 8; NSAID, nonsteroidal anti-inflammatory drugs; PDGF, platelet-derived growth factor; SFM, serum-free medium; TGF-α, transforming growth factor α; TGF-β, transforming growth factor β; VEGF, vascular endothelial growth factor.
